# Controlling the narrative: the relationship between narrative ability and executive functioning in children with developmental language disorder

**DOI:** 10.3389/fpsyg.2024.1489997

**Published:** 2024-11-15

**Authors:** Lonneke Janssen, Annette Scheper, Constance Vissers

**Affiliations:** ^1^Royal Dutch Kentalis, Sint-Michielsgestel, Netherlands; ^2^Behavioural Science Institute, Radboud University, Nijmegen, Netherlands

**Keywords:** narrative ability, executive functioning, developmental language disorder, children, neuropsychology

## Abstract

Children with developmental language disorder (DLD) experience problems in language comprehension and/or production. In particular, storytelling or narrative ability is often impaired, as this type of discourse involves all domains of language. These problems may lead to a lower quality of social interaction and mental health. Moreover, problems in oral narrative ability during early development have a negative effect on later literacy. However, telling a story involves more than language alone. Executive functioning is thought to play an important part in stimulating narrative ability, as linguistic utterances need to be planned in a temporal and causal order, and switching is needed between multiple characters and events in the story. Research has shown that children with DLD experience problems with executive functioning, independent of their language ability. Thus, the difficulties in storytelling may be caused by both impaired language and executive functioning, as both domains follow hierarchical developmental paths during the early childhood years. In this review, we discuss three components of narrative ability (comprehension, production of macrostructure and production of microstructure) and how they may be interconnected to the three core components of executive functioning (working memory, switching and inhibition) and attention. This review shows that updating and monitoring information in working memory plays an important part in all three components of narrative ability, across multiple studies. This result may give direction in the development of narrative assessment and intervention, and urge further research to disentangle the interplay between language and executive control in DLD.

## 1 Introduction

### 1.1 Narrative ability and executive functions

Narration is a competence that has communicative, social and academic importance. To tell a coherent story children have to learn to produce series of utterances which are temporally, logically and causally related (Berman and Slobin, 1994). Young children are quickly expected to demonstrate their narrative ability by talking about their experiences, feelings and life events in social settings. In language development, narrative ability can be seen as the most complex linguistic task, as children need to integrate rich vocabulary into grammatical utterances that vary in complexity. Moreover, utterances must be cohesive and coherent. Singular utterances in successive order must form a logical structure to communicate appropriately. Executive functions (EF) are thought to play an important role in narration (Büttner, 2016). EF are defined as ‘general-purpose control mechanisms that modulate the operation of various cognitive subprocesses and thereby regulate the dynamics of human cognition’ (Miyake et al., 2000, p. 50). EFs are regarded as top-down processes that control and regulate goal-oriented behavior needed in day-to-day activities. Miyake et al. (2000, p. 50) identified three core components of EF: the ability to switch flexibly between different tasks (switching), update and monitor working memory (WM), and ignore distracting information or suppress automatic responses (inhibition). According to Büttner (2016), the prerequisites for comprehension and production of narratives are related to specific EFs. These relationships include the causal-temporal ordering of events (updating and monitoring), taking into account the mental states of different characters and switch perspectives between them (switching), and suppressing redundant details or other characters’ perspectives (inhibition).

EF and language (e.g., narrative skills) develop concurrently and it is theorized that the development of language and EF occurs hierarchically. The development of the core EFs are preceded by the development of sustained attention, which starts at infancy and is defined as the ability to focus on a specific target for prolonged time periods (Garon et al., 2008; Marini et al., 2020). The core components of EF start developing before 3 years old but reach a critical period between 3 and 5 years old (Garon et al., 2008). The ability to keep information active in WM and inhibitory control predict shifting, which suggests that shifting is a more complex EF ability that builds on other EF abilities such as WM and inhibition (Garon et al., 2014). The development of narrative skills follows a similar path from simple to more complex structures. Narratives at 2 years old consist of descriptions of character actions without a central theme (Scionti et al., 2023). From 3 to 4 years, children include causal connections between these actions. But it is not until 6 or 7 years old that children use “true coherent narratives,” in which utterances are temporally, logically and causally related. However, this continues to develop until at least 10 years old (Scionti et al., 2023).

Research shows that if the development of different complex abilities such as language and EF happens concurrently, interaction takes place between functions (facilitation of inhibition) (Bernstein and Waber, 2018; Nilsson and de López, 2016; Carlson et al., 2002; Vissers et al., 2015). In the case of EF and narrative skills, the ability to keep information active in WM might facilitate connecting different events in the story temporally and causally, later supplemented with linking them to a central theme.

### 1.2 Developmental language disorder

The development of narrative skills does not occur without effort for all children, however. Children with developmental language disorder (DLD) have difficulty acquiring and using language (Bishop et al., 2017). DLD is defined as a neurocognitive developmental disorder that presents itself primarily in the language domain, such as in comprehension, morpho-syntax, semantics, phonology or pragmatics (e.g., narrative ability). The difficulties in acquisition and production of language may not be explained through any intellectual, biomedical or severe hearing impairment (Bishop et al., 2014). The narratives of children with DLD are shorter, show problems in complexity, grammaticality, coherence and fluency and contain a lower information or plot value compared to the narratives of typically developing (TD) peers (Bergmann et al., 2017; Bliss and Pierre, 1997; Christensen, 2019; Cleave et al., 2010; Colozzo et al., 2011; Duinmeijer et al., 2012; Guo et al., 2008; Pearce et al., 2010; Rodríguez et al., 2017).

Moreover, research has shown that the difficulties observed in children with DLD are not limited to the language domain, but also include difficulties in the executive domain, for instance in phonological and visuospatial WM, cognitive flexibility, and inhibition, even while controlling for language ability (Pauls and Archibald, 2016; Vissers et al., 2015; Vugs et al., 2013, 2017).

### 1.3 The current literature review

Tomas and Vissers (2019) proposed a model for the developmental interconnections between language and other higher cognitive functions. According to them, the interplay between perception, attention, EF and language might underlie the observed problems in communication in children with DLD (Tomas and Vissers, 2019). Through the observed problems in both domains, and the fact that development of these domains happens concurrently and may be facilitated or inhibited by each other, it could be hypothesized that this interconnection is present for narrative ability and EF.

In the current review, we zoom in on the literature regarding the relationship between narrative ability and EF through peer reviewed experimental research on children with DLD aged 4–7 years old. In a recent meta-analysis conducted for TD children and adolescents, it was found that narrative ability and EF are weakly associated (Scionti et al., 2023). However, they also found that the associations between narrative ability and EF are stronger in children with atypical development between 3 and 7 years old than for their TD peers. Atypical development included children with different neurocognitive developmental disorders, including DLD. This highlights the need for differentiation in the way we investigate these problems in children with and without developmental difficulties. The weaker narrative ability of children with DLD in particular may have a negative impact on their social and academic development. Their participation in daily communication may be less frequent and of a lesser quality. Indeed, children with DLD show lower social–emotional well-being (Goh et al., 2021; Maggio et al., 2014). Moreover, narrative competence contributes to academic success, because adequate oral narrative ability predicts later reading and writing ability (Reese et al., 2010; Schaughency et al., 2017). Academic success is often lower for children with DLD (Conti-Ramsden, 2008). Identification of specific interconnections between narrative ability and EF may inform clinical practice to make more evidence-based assessment, intervention decisions and recommendations.

In this review, narrative ability is divided into three components: narrative comprehension, production of macrostructure (story-level) and production of microstructure (sentence-level). For EF, we will discuss the three core components identified by Miyake et al. (2000). We also include attention, as controlled attention is taken as a common requirement in executive tasks and constitutes the first step in the hierarchical development of EF (Garon et al., 2008, 2014; Miyake et al., 2000). In Table 1 the available literature regarding the relationship between narrative ability and EF is presented. For each study, the investigated domains and methods are described in a detailed overview.

**Table 1:** Overview of discussed papers with children’s age, method of narrative and cognitive assessments and their scoring.

Paper	Age range M (SD in months)	Assessment of executive functioning or attention	Domains of executive functioning or attention	Assessment of narrative ability	Narrative comprehension scoring	Narrative macrostructure scoring	Narrative microstructure scoring
Dawes et al. (2018)	5;7 (range 5;2-6;2)	1. Comprehensive test of Phonological Processing (Wagner et al., 1999) 2. Repeating Sentences task of the Test of Language Development – Primary, Third edition (Hamill and Newcomer, 1997) 3. Bear/dragon task and grass/snow task (Carlson, 2005)	1. Working memory – phonological loop 2. Working memory – episodic buffer 3. Inhibition	Squirrel Story Narrative Comprehension Assessment (Dawes et al., 2006)	Comprehension questions with pictures as reference	Retelling: story structure and story content rating	Retelling: level of language, syntax and vocabulary ratings
Dicataldo et al. (2023)	6;6 (3.7)	1. Digit span task of the WISC-IV (Wechsler, 2003) 2. The Day & Night Test (Usai et al., 2017) 3. Modified Wisconsin Card Sorting Test (Cianchetti et al., 2007)	1. Verbal working memory 2. Inhibition 3. Cognitive flexibility	Test for Listening Comprehension (Levorato and Roch, 2007)	Comprehension questions regarding explicit and implicit information	Not applicable (NA)	NA
Dodwell and Bavin (2008)	6;7 (3)	1. The Number Recall of the Kaufman Assessment Battery for Children (Kaufman and Kaufman, 1983) 2. Word list recall (Adams et al., 1999) 3. Central executive task (Gaulin and Campbell, 1994) 4. Recalling sentences of the CELF-3 (Semel et al., 1995) 5. Auditory Continuous Performance Test (Hanson and Montgomery, 2002)	1. Working memory – phonological memory span 2. Phonological memory 3. Working memory – central executive 4. Working memory – episodic buffer 5. Sustained attention	1. The Birthday Story (BS; Culatta et al., 1983) 2. ERRNI (Bishop, 2004)	1. BS: Comprehension questions invoking literal information and inferencing 2. ERRNI: Comprehension questions invoking literal information and inferencing	ERRNI (generation and recall): total amount of listed story ideas (max. 24 ideas)	NA
Duinmeijer et al. (2012)	7.35 (1.05)	1. Sustained attention subtest (TEA-Ch, Manly et al., 1998) 2. Digit span task of the WISC, Dutch version (Kort et al., 2005) 3. Word list recall task, a Dutch version of the California Verbal Learning Test, Children’s version (Kalverboer and Deelman, 1964)	1. Sustained attention 2. Verbal working memory 3. Verbal working memory	1. Retelling: Bus Story Test (Renfrew, 1997; Jansonius et al., 2014) 2. Generation: Frog Story Test (Mayer, 1969)	NA	1. Retelling: plot score (max. 25 elements) 2. Generation: plot score (max. 19 elements)	Retelling and generation: mean length of utterance, mean length of the five longest utterances, grammatical accuracy, syntactic complexity, non-fluency
Kalliontzi et al. (2022)	4;5 (2.2)	1. Verbal and non-verbal n-back type task (Yang and Gray, 2016) 2. Verbal and non-verbal Flanker type task (Yang and Gray, 2016) 3. Verbal and non-verbal sorting card task (Yang, 2015)	1. Updating working memory 2. Inhibition 3. Cognitive flexibility	Logometro (Mouzaki et al., 2017)	Comprehension questions	Generation and retelling: score for the event, the problem and the potential solution	Generation and retelling: references in the agents
Marini et al. (2020)	5.19 (0.03)	1. Forward and backward digit recall test of the Wechsler Scales (Wechsler, 1993) 2. Developmental Neuropsychological Assessment (Urgesi et al., 2011)	1. Updating verbal working memory 2. Inhibition	Battery for the assessment of language in children aged 4 to 12 (BVL 4-12; Marini et al., 2015)	NA	NA	Lexical informativeness and global coherence
Smolak et al. (2020)	7;3 (range 6;0 – 8;0)	1. Track-it Task (Erickson et al., 2015; Fisher et al., 2013) 2. Odd One Out task (Henry, 2001)	1. Sustained attention 2. Visuo-spatial working memory	Test for Narrative Language, edition 1 or 2 (Gillam and Pearson, 2004, 2017)	Comprehension questions	Generation: producing key thematic elements	NA

## 2 Narrative comprehension and EF

The comprehension of narratives is usually measured as the ability to correctly answer comprehension questions about a model story. These questions can refer to explicit and implicit information. Children either recall explicit information from the story or are required to integrate information in the story with general, real-world knowledge that is implicit (Dicataldo et al., 2023).

### 2.1 Narrative comprehension and attention

Evidence that confirms a relationship between comprehension and attention was not found. To the contrary, Smolak et al. (2020) found that sustained attention did not correlate significantly with narrative comprehension. Sustained attention was measured by a tracking task with two conditions: a homogeneous and heterogeneous condition, where in the latter the distractor shapes are all the same but different from the target shape, and in the former all distractor shapes are different. Children with DLD performed worse than TD in both conditions, the homogeneous condition did not facilitate tracking of the target shape. However, the ability to track and identify target shapes was not correlated with narrative comprehension for children with DLD.

### 2.2 Narrative comprehension and working memory, switching and inhibition

The relationship between narrative comprehension and updating and monitoring in WM has been researched intensely. More often than not, associations are found between narrative comprehension and WM. Comprehension and WM are related in digit or word span tasks and non-word repetition tasks, which are seen as verbal measures (Dawes et al., 2018; Dicataldo et al., 2023; Dodwell and Bavin, 2008). The relationship between verbal measures of EF and comprehension in children that have difficulty in language development seems unsurprising. However, the relationship is also present where the WM task is non-verbal in nature (Kalliontzi et al., 2022). In this particular study a relationship was found between visuo-spatial WM and narrative comprehension in younger children with DLD, highlighting that language problems are related to more domain-general cognitive functions as well. Importantly, non-verbal visuo-spatial WM predicted narrative comprehension (along with switching) with an explained variance of 21%.

Moreover, a relationship exists between narrative comprehension and switching in children with DLD (Dicataldo et al., 2023; Kalliontzi et al., 2022). Importantly, both of these studies not only found correlations between both domains, but also that switching was a predictor for narrative comprehension. In Kalliontzi et al. (2022) this predictor was significant alongside visuo-spatial WM.

Narrative comprehension also seems to relate to inhibition (Kalliontzi et al., 2022). In this study, children needed to push a button according to the direction the middle fish in a row of five fish was looking in. There was a congruent condition (all fish were looking in the same direction) and an incongruent condition (the middle fish was looking in a different direction). It was found that increased reaction times on this inhibition task were associated with more incorrect comprehension questions for children with DLD (*r* = −0.29).

## 3 Narrative macrostructure and EF

Narrative macrostructure can be defined as the global organization or structure of a story, wherein it is important to establish coherence between sentences and in the narrative as a whole. The way narrative macrostructure is operationalized varies between studies. Some studies use story grammar (Stein and Glenn, 1979). Here, narrative macrostructure includes producing the key story grammar elements such as introduction of the main character, setting, problem, internal emotional response, actions and the resolution. However, some studies use other measures, such as errors in coherence or referents, and informativeness (based on lexical diversity).

### 3.1 Narrative macrostructure and attention

Even though a relationship was not found for comprehension, sustained attention does play a role in producing narrative macrostructure (Duinmeijer et al., 2012; Smolak et al., 2020). Duinmeijer et al. (2012) measured narrative macrostructure in two narrative genres (mean age 7.4 years). Story retelling was measured through the *Bus Story Test* (Bolk and Scheper, 2024; Jansonius et al., 2014; Renfrew, 1997) and story generation through the *Frog Story Test* (Mayer, 1969; Scheper and Blankenstijn, 2013). To measure sustained attention, children had to count the number of sounds they heard silently until the end of the item. It was found that sustained attention correlated significantly with narrative macrostructure (i.e., the number of story grammar elements produced) in the story generation task, but not in the story retelling task.

Smolak et al. (2020) used a composite score of both narrative macro- and microstructure to investigate the relationship with attention. Narrative macrostructure was scored as producing key thematic elements. The ability to track the target shape in both conditions (homogeneous and heterogeneous) was associated with narrative language production as a whole.

### 3.2 Narrative macrostructure and working memory, switching and inhibition

There is evidence for a relationship between macrostructure and WM in children with DLD (Dodwell and Bavin, 2008; Duinmeijer et al., 2012; Marini et al., 2020), similar to the relationship found for narrative comprehension. In all three studies, the WM tasks were verbal, namely a retelling sentences task, word list recall and digit recall. It seems that keeping information active and being able to manipulate this information in WM is important in producing narrative macrostructure.

Moreover, a relationship exists between macrostructure and switching (Kalliontzi et al., 2022). It was found that macrostructure in a self-generated narrative correlated significantly with verbal switching, and in the retelling task with non-verbal switching. The difference between the two switching tasks was small, however. To assess verbal switching, children performed a sorting task in which they saw images of scissors on a red background and glasses on a blue background. The child had to sort cards by object or color by pushing a button on a keyboard (Kalliontzi et al., 2022, p. 5). In the non-verbal condition the only difference was that the images of the scissors and glasses were replaced with images of polygons on two different bags and children were asked to perform the same sorting task. Both tasks did not need the child to give any verbal response to an item.

Evidence has been found for a relationship between narrative macrostructure and inhibition as well (Marini et al., 2020). Lexical informativeness correlated significantly with the inhibition measure, in which children first named the shapes of circles and squares or the up and down direction of arrows (naming condition). In the inhibition condition, children were asked to provide the opposite naming response on the same stimuli. However, it can be argued whether lexical informativeness is an adequate measure for narrative macrostructure, as it relates to informativeness on the word level, instead of the global organization or structure of a story as a whole.

## 4 Narrative microstructure and EF

Narrative microstructure relates to the within-sentence level. It can be operationalized in a variety of ways. Studies may measure the mean length of utterances (MLU), the level of grammatical accuracy, embedding of subordinate clauses (a measure for syntactic complexity), fluency of utterances, use of conjunctions and so on. Whether microstructure is measured with just a single variable or multiple, varies between studies.

### 4.1 Narrative microstructure and attention

A relationship between narrative microstructure and attention exists (Duinmeijer et al., 2012; Smolak et al., 2020). In Duinmeijer et al. (2012), the mean length of utterances (MLU) and the mean length of the five longest utterances (MLU5) correlated significantly with sustained attention in the story retelling task. A discrepancy exists between the two narrative genres that were investigated, because in story generation, MLU and MLU5 did not correlate with sustained attention, but rather with WM. Evidently, both genres place a different demand on cognitive functions. In story retelling, children were asked to listen to the model story first, which puts a higher demand on sustained attention and is therefore needed in producing longer utterances.

As discussed previously, Smolak et al. (2020) investigated the relationship between attention and narrative language production as a whole. Narrative microstructure was measured by counting causal conjunctions (e.g., *because, as, since*), grammatical accuracy and character dialogue. Narrative language production, which included these microstructural variables, correlated significantly with sustained attention.

### 4.2 Narrative microstructure and working memory, switching and inhibition

There is evidence for a relationship between narrative microstructure and WM (Duinmeijer et al., 2012). Emphasizing once more the difference between narrative genres, MLU and MLU5 in the story generation task correlated significantly with digit recall (forward and backward), and MLU5 correlated significantly with word list recall as well. The ability to recall digits and manipulate the order in which to recall them plays a role in producing longer utterances. Moreover, MLU5 is an indirect measure of complexity, as complex utterances are often longer than simple ones. It seems that the ability to remember words is more important for producing even longer and possibly more complex sentences.

Kalliontzi et al. (2022) found a relationship between ‘narrative language’ (macro- and microstructure taken together) and switching in both story retelling and story generation.

## 5 Discussion

In this review, we discussed the evidence for interconnections between narrative comprehension and production, three core components of EF (updating and monitoring in WM, switching and inhibition) and attention in children with DLD. The evidence points to an important role of WM in narrative comprehension, macrostructure and microstructure. The ability to hold, update and manipulate information in WM is involved in understanding narratives, in creating a global coherent organization and in producing longer, and indirectly, more complex utterances in narratives (Dawes et al., 2018; Dicataldo et al., 2023; Dodwell and Bavin, 2008; Duinmeijer et al., 2012; Kalliontzi et al., 2022; Marini et al., 2020). A relationship between narrative ability and switching or inhibition was less clear-cut. In any case, Dicataldo et al. (2023) and Kalliontzi et al. (2022) did find that switching was a significant predictor for narrative comprehension. This provides some evidence of the role of switching besides WM in narrative comprehension.

We did not find evidence for relationships between all aspects of EF and narrative ability. The literature on narrative microstructure is sparse. It seems that narrative macrostructure is more often the topic of research interest. This is understandable as the unique characteristic of a story is the global organization of coherent language into a unified construct, with a beginning, middle and end. However, the ability to produce grammatical and complex utterances is a prerequisite to produce adequate macrostructure in a narrative, for instance through the use of obligatory syntactic arguments, conjunctions and embedded clauses.

As we have found evidence for a relationship between narrative ability and updating and monitoring information in WM in children with DLD aged 4–7 years, it is also important to understand how these relationships change over time. As Scionti et al. (2023) found in their meta-analysis, the relation between narrative ability and EF in atypical development (which included DLD) was stronger in the ages 3–7 years than in TD children of the same age. Importantly, after 7 years old, effect sizes between both groups did not differ. Across all children with (a)typical development, it was found that relationships between narrative ability and EF decrease from age seven and up. This may be related to the finding that EF components reach a critical development period between 3 and 5 years old, while the development of narratives is still underway, and continues to until 10 years of age (Garon et al., 2008; Scionti et al., 2023). Perhaps other cognitive and environmental factors influence language development at this developmental stage. In a recent review by Smit et al. (2022), it was stated that children aged 6–12 years old learn the ability “to predict not only what someone else thinks or feels, but also what another person thinks of how someone else feels or thinks” (i.e., second-order theory of mind (ToM); Smit et al., 2022, p. 2). This ability may be important for perspective-taking in telling narratives. Indeed, in two of the articles discussed in this review, ToM seemed to play a role, at least in narrative comprehension. In Dawes et al. (2018), ToM correlated stronger with comprehension than WM. Moreover, in Dicataldo et al. (2023), verbal ToM predicted narrative comprehension. The influence of cognitive factors other than EF must be taken into account in researching theoretical underpinnings of (a)typical language development. With respect to Vissers and Tomas’ neuropsychological approach to DLD, to reach better informed decisions in narrative intervention, we need to take a broader perspective than the linguistic domain alone. Tapping into the interplay between EF development (WM in particular) and language development (narrative ability in particular) while assessing and treating children with DLD might lead to more academic success and more meaningful social relationships.
